# Exercise intolerance in patients with chronic coronary syndrome: insights from exercise stress echocardiography

**DOI:** 10.3389/fcvm.2024.1442263

**Published:** 2024-11-28

**Authors:** Wei-wei Zhu, Run-Yu Tian, Di-chen Guo, Ming-ming Lin, Qi-zhe Cai, Yun-yun Qin, Xue-yan Ding, Xiu-zhang Lv

**Affiliations:** Department of Echocardiography, Beijing Chao Yang Hospital, Capital Medical University, Beijing, China

**Keywords:** chronic coronary syndrome, exercise stress echocardiography, exercise intolerance, stroke volume, oxygen consumption

## Abstract

**Aims:**

This study applied exercise stress echocardiography (ESE) to identify risk factors associated with exercise intolerance in patients with chronic coronary syndrome (CCS).

**Methods and results:**

90 CCS patients underwent a cardiopulmonary exercise test and ESE, assessing exercise capacity, left ventricular systolic and diastolic function, and systolic reserve. The patients were divided into two groups according to the percentage of predicted oxygen consumption (VO_2_) at peak (≥85%, normal exercise tolerance group; <85%, exercise intolerant group). The left ventricular ejection fraction, average mitral valve S’, and left ventricular global longitudinal strain were lower in the exercise intolerant group than in the normal group, but no significant differences were observed in myocardial work parameters at rest. The average mitral valve E/e’, EDVi/E/e’, and proportion of abnormal diastolic function at the peak were higher in the exercise intolerant group than in the normal group. Moreover, the *Δ*SVi and flow reserve were lower, but the *Δ*average mitral valve E/e’ was higher in the exercise-intolerant group. From univariate and multivariate logistic regression analysis, only peak EDVi/E/e’ and *Δ*SVi correlated independently with exercise intolerance in CCS patients. With cutoff values of 8.64 ml/m^2^ for peak EDVi/E/e’ and 12.17 ml/m^2^ for *Δ*SVi, the combination of these factors had an area under the receiver operating characteristic curve of 0.906 (95% confidence interval, 0.820–0.960) for the prediction of exercise intolerance in CCS patients.

**Conclusion:**

Hemodynamic changes during exercise in CCS patients were effectively evaluated using ESE. An elevated peak EDVi/E/e’ and a decreased *Δ*SVi are independent risk factors for exercise intolerance in patients with CCS.

## Introduction

Coronary artery disease (CAD) is one of the leading causes of death and morbidity worldwide and imposes high societal and economic burdens (1). The dynamic nature of CAD progression results in various clinical presentations that are generally categorised as either acute coronary syndrome (ACS) or chronic coronary syndrome (CCS). CCS is chronic, most often progressive, and hence severe, even in clinically apparently silent periods (2). Therefore, effective clinical management of these patients is critical.

Exercise intolerance is a common early symptom in patients with CCS and may be related to myocardial ischemic injury caused by coronary artery stenosis, left ventricular systolic and diastolic dysfunction, functional mitral regurgitation, chronotropic insufficiency, and peripheral muscle dysfunction (3). Previous studies have found that exercise intolerance can be associated with short-term and long-term adverse cardiovascular outcomes in patients with CCS (4, 5). However, the specific pathophysiological mechanism of exercise intolerance in patients with CCS remains unclear. Cardiopulmonary exercise testing (CPET) is a non-invasive, reliable tool for estimating global oxygen consumption (VO_2_), which can effectively reflect exercise capacity (6). However, CPET offers a limited ability to differentiate the responsible mechanisms when an abnormally low value is detected (7). Combining resting or stress echocardiography with CPET provides a far more informative approach, giving insights into the morphological and functional cardiac issues that may impact exercise capacity (8). To date, exercise stress echocardiography (ESE) has been rarely used to analyze the mechanism of exercise intolerance in patients with CCS. Our study aimed to evaluate the exercise capacity of CCS patients by CPET and explore whether ESE parameters can be used to predict exercise intolerance in these patients as well as to analyze its possible pathophysiological mechanism.

## Methods

### Study population

We prospectively enrolled 90 patients aged >18 years suspected of having CCS between September 2021 and May 2023 in our cardiology department. These patients visited our cardiology department due to chest discomfort (chest pain, tightness) or shortness of breath at rest or after activity. CCS was defined according to the 2019 European Society of Cardiology guidelines (2). The inclusion criteria were as follows: suspected CAD and “stable” anginal symptoms or dyspnea and evaluation by coronary computed tomography angiography (CCTA); and prior treatment with coronary artery intervention and stable hemodynamics for at least 1 month. The selected populations were classified into the I, III, and IV clinical phenotypes of CCS. All patients were able to perform bicycle exercise. The exclusion criteria were as follows: recent occurrence of acute myocardial infarction, history or presence of symptomatic congestive heart failure, Left ventricular ejection fraction <40%, a respiratory exchange ratio (RER) <1.0 at peak on CPET, anaemia, decompensated thyroid disease, chronic kidney disease, chronic lung disease, severe valvular disease, hypertrophic cardiomyopathy with left ventricular outflow tract obstruction, resting echocardiography suggesting pulmonary hypertension, inadequate acoustic window for echocardiography, and significant arrhythmias at rest. We collected data on demographic and clinical characteristics as well as from CCTA by querying electronic medical records, and Dr. Mingming Lin and Dr. Yunyun Qin recorded all data. The physicians who performed CPET (Dr Xi-yan Yang, Dr Wen-shu Zhao, and nurse Nian-Zhong Zhang) and ESE (Dr Weiwei Zhu and Dr Runyu Tian) were blind to the clinical data and were also blinded to the respective results.

The study was conducted in according with the Declaration of Helsinki. The institutional review board of Beijing Chaoyang Hospital, Affiliated with Capital Medical University, approved the study protocol, and all participants provided written informed consent.

### Baseline and exercise stress echocardiography

All patients underwent a comprehensive transthoracic echocardiography examination at rest according to the latest guidelines (9), using a commercially available ultrasound system (Vivid E95, GE Healthcare, Horton, Norway). A standard multistage, symptom-limited, semi-supine exercise test was performed using a bicycle ergometer (Ergoselect 1200, Stress EchoCouch Ergometer; Ergoline, Bitz, Germany) after the resting echocardiography (10). Participants were encouraged to complete exercise to their peak (when they achieved 85% of the age-predicted maximum heart rate) or until exhaustion if someone took beta-blockers. We acquired exercise echocardiographic images at two stages: low-load effort (within the first 4 min of exercise) and peak effort. We summarized the conventional data acquisition protocol in Supplementary Table S1. All patients completed the parameter measurements in Supplementary Table S1 and underwent LV diastolic function evaluation according to the 2016 American Society of Echocardiography guidelines (11). We calculated the LVEDV/E/e′ ratio as a surrogate of LV compliance during exercise and LVEDVi/E/e′ ratio obtained after body surface area normalization (12). Flow reserve was defined as an increase in stroke volume (SV) from rest to peak effort [(SV_peak_−SV_baseline_)/SV_baseline_] (13). Offline speckle-tracking echocardiography analysis included global longitudinal strain (GLS) and myocardial work analysis with evaluation of the myocardial work parameters global work index (GWI), global constructive work (GCW), global wasted work (GWW), global work efficiency (GWE) while ensuring a frame rate >50 Hz at rest. We analyzed all echocardiographic data offline using dedicated software (EchoPAC version 204, GE Healthcare). All parameters were measured according to the recommendations of the American Society of Echocardiography (9).

### Cardiopulmonary exercise test

All patients underwent a standard symptom-limited graded ramp bicycle exercise test in the upright position according to guideline (7) with continuous monitoring of a 12-lead electrocardiogram, brachial blood pressure, and non-invasive arterial saturation. The parameters analyzed included the peak oxygen consumption (VO_2_),%pred VO_2_ at peak, RER at peak, O_2_ pulse at peak, VO_2_ at anaerobic threshold (AT), ventilatory efficiency (VE/VCO_2_ slope), oxygen uptake efficiency slope (OUES) and heart rate (HR) for the first 1 min of recovery. The anaerobic threshold was calculated using a dual method approach (V-slope and ventilatory equivalent methods). OUES was derived from the relationship between VO_2_ (plotted on the *y*-axis) and the log transformation of VE (*x*-axis). The arteriovenous oxygen difference (A-VO_2_Diff) was calculated using the Fick equation: VO_2_/cardiac output calculated from echocardiography (13, 14). The interval between the cardiopulmonary exercise test and ESE was more than 24 h but less than seven days. With reference to the 2022 Chinese expert consensus and the 2012 EACPR/AHA joint scientific statement, we divided patients into two groups according to the percentage of predicted VO_2_ at peak (15, 16): those with normal exercise capacity based on ≥85% predicted VO_2_ at peak (normal group) and those with reduced exercise capacity based on <85% predicted VO_2_ at peak (exercise intolerant group).

### Statistical analyses

We performed statistical analysis using SPSS Version 20 for Windows (IBM, Armonk, NY, USA) and MedCalc Version 15.6.1 (MedCalc Software, Ostend, Belgium) statistical software. Continuous variables are expressed as mean ± standard deviation values or median values and interquartile ranges where appropriate. Categorical data are presented as absolute numbers or percentages. Two-sample comparisons were performed using the *t*-test if variables were normally distributed or the Mann–Whitney *U*-test if variables were not normally distributed. Categorical data were compared using the chi-squared or Fisher's exact test. Either the Pearson or Spearman correlation coefficient was used to analyze the linear correlation between echocardiographic parameters, peak oxygen consumption, and %pred VO_2_ at peak. We used univariate or multivariate stepwise logistic regression to identify independent risk factors associated with exercise intolerance in patients with CCS. The C-statistic was used to evaluate the predictive value of the model. Receiver operating characteristic (ROC) curve analysis was used to calculate the area under the curve and the cutoff point with the highest Youden index.

The same observer and an independent reader reanalyzed a random sample of 20 patients. We measured the reproducibility of the variables, including left ventricular end-diastolic volume (LVEDV), left ventricular end-systolic volume (LVESV), SV, wall motion score index (WMSI) at rest, and during any stage of the exercise, and LVGLS at rest, by calculating intraclass correlation coefficients and coefficients of variation. All tests were two-sided, with a *P* value of <.05, indicating statistical significance.

## Results

### Baseline characteristics

Table 1 presents the demographic and clinical characteristics of the study population. Compared with the group with normal tolerance, the exercise-intolerant group showed a younger age, higher proportions of males and smokers, a larger body surface area (BSA), and a higher prevalence of previous percutaneous coronary intervention (PCI). No significant differences in blood test results or therapies were observed between the groups, and CCTA showed no difference in the proportion of single vessel stenosis >50% between the patient groups.

**Table 1:** Demographic and clinical characteristics for patients with CCS according to the percentage of predicted oxygen uptake at peak (normal exercise capacity group, ≥85%; exercise intolerant group, <85%).

Variables	Total (*n* = 90)	Normal group (*n* = 36)	Exercise intolerant group (*n* = 54)	*P* value
Demographic characteristics				
Age, years	50.13 ± 10.68	54.86 ± 11.23	46.98 ± 9.11	<0.001
Sex, male	54 (60%)	14 (39%)	40 (74%)	0.001
BSA, m^2^	1.82 ± 1.89	1.76 ± 0.17	1.87 ± 0.19	0.006
SBP, mmHg	119.03 ± 13.91	119.78 ± 12.64	118.54 ± 14.79	0.681
DBP, mmHg	72.58 ± 11.17	70.11 ± 10.22	74.22 ± 11.57	0.087
HR, beats/minute	71.13 ± 8.6	69.69 ± 9.53	72.09 ± 7.86	0.197
Smoker	27 (30%)	6 (17%)	21 (39%)	0.024
Clinical characteristics				
AH	30 (33%)	12 (33%)	18 (33%)	1.000
Diabetes	6 (7%)	2 (6%)	4 (7%)	0.730
Dyslipidemia	46 (51%)	19 (53%)	27 (50%)	0.796
A single vessel stenosis rate ≥50% in CCTA	19 (21%)	5 (14%)	14 (26%)	0.170
Previous PCI	15 (17%)	2 (6%)	13 (24%)	0.021
Blood tests				
CKMB, ng/ml	0 (0, 0.82)	1.2 (0.8–1.2)	2.0 (1–2)	0.700
cTn, ng/ml	0 (0,0)	0.04 (0.01–1.10)	0.03 (0.03–0.04)	0.858
BNP, pg/ml	42 (26,62)	21 (11–134)	26 (16–89)	0.866
Total cholesterol, mmol/L	4.47 ± 0.86	4.63 ± 0.89	4.33 ± 0.82	0.176
LDL, mmol/L	2.60 ± 0.96	2.83 ± 0.96	2.44 ± 0.93	0.065
HDL, mmol/L	1.18 ± 0.36	1.26 ± 0.33	1.12 ± 0.37	0.071
Triglycerides, mmol/L	1.49 (1.06–2.03)	1.77 ± 1.01	0.85 (0.80–1.53)	0.336
Therapy				
Beta-blocker	12 (13%)	5 (14%)	7 (13%)	1.0
CCB	16 (18%)	7 (19%)	9 (17%)	0.736
ACEi or ARB	12 (13%)	2 (6%)	10 (18%)	0.145
ASA	25 (28%)	10 (28%)	15 (28%)	1.0
Oral anticoagulant	14 (16%)	5 (14%)	9 (17%)	0.722
Statins	44 (49%)	18 (50%)	26 (48%)	0.863
Oral hypoglycemic drugs	6(7%)	2(6%)	4(7%)	1.0

BSA, body surface area; SBP, systolic blood pressure; DBP, diastolic blood pressure; HR, heart rate; AH, arterial hypertension; CCTA, coronary computed tomography angiography; PCI, percutaneous coronary intervention; CKMB, creatine kinase isoenzymes; cTn, cardiac troponin; BNP, brain natriuretic peptide; LDL, low-density lipoprotein; HDL, high-density Lipoprotein; CCB, calcium channel blocker; ACEi, angiotensin-converting enzyme inhibitor; ARB, angiotensin II receptor blocker; ASA, acetylsalicylic acid.

### Combined stress echocardiography and cardiopulmonary exercise testing

We conducted the cardiopulmonary exercise test and exercise stress echocardiography in two separate sessions due to the limitations of the equipment. 34% of patients reached the diagnostic endpoints for ESE (85% of age-predicted maximal heart rate). The reasons for test interruption were dyspnea (8%), fatigue (23%), leg discomfort (28%) and high blood pressure [systolic blood pressure (SBP) ≥ 250 mmHg, 7%]. No patient experienced persistent chest pain and/or syncope. Supplementary Table S2 shows a comparison of the two testing protocols.

The results for cardiopulmonary parameters are compared between the groups in Table 2. Each patient made a maximal effort during the exercise, and the mean RER at peak was 1.21. The exercise-intolerant group completed a lower workload, with a mean predicted workload at a peak of 78 W vs. 102 W in the normal group. The mean VO_2_ at peak in the exercise intolerant group was 20.99 ml/kg/min, which also was lower than that in the normal group (24.11 ml/kg/min), but the exercise intolerant group had a relatively preserved VO_2_ (peak VO_2_ > 20 ml/kg/min).

**Table 2:** Cardiopulmonary parameters for patients with CCS according to the percentage of predicted oxygen uptake at peak (normal exercise capacity group, ≥85%; exercise intolerant group, <85%).

Variables	Total (*n* = 90)	Normal group (*n* = 36)	Exercise intolerant group (*n* = 54)	*P* value
Workload, W at peak	123 ± 41	118 ± 45	125 ± 39	0.435
Workload,% predicted at peak	87.55 ± 18.46	101.58 ± 13.33	78.20 ± 15.24	<0.001
VE/VCO_2_ at peak	30.45 ± 5.73	30.06 ± 7.48	30.72 ± 4.26	0.592
VO_2_, ml/kg/minute at peak	22.24 ± 4.94	24.11 ± 5.90	20.99 ± 3.74	0.007
VO_2_,% predicted at peak	80.86 ± 15.41	95.69 ± 10.47	70.96 ± 8.77	<0.001
RER at peak	1.21 ± 0.13	1.16 ± 0.09	1.24 ± 0.14	0.003
O_2_ pulse, ml/beat at peak	11.53 ± 3.07	11.93 ± 3.65	11.27 ± 2.61	0.320
HR, beats/minute at peak	140 ± 19	141 ± 19	139 ± 19	0.646
SBP, mm Hg at peak	173 ± 23	176 ± 22	170 ± 24	0.248
DBP, mm Hg at peak	85 ± 10	82 ± 11	86 ± 9	0.040
VO_2_, ml/kg/minute at AT	13.06 ± 3.21	14.45 ± 3.94	12.15 ± 2.23	0.003
VE/VCO_2_ slope	27.64 ± 5.18	27.11 ± 3.66	28.00 ± 5.99	0.429
OUES, ml/kg/min	1,596.67 ± 435.99	1,650.87 ± 503.23	1,560.54 ± 385.85	0.352
HR recovery, beats/minute	21 ± 8	20 ± 9	21 ± 7	0.616
A-VO_2_Diff, ml/dl at peak	17.72 ± 3.85	18.41 ± 3.97	17.24 ± 3.74	0.170

VE, minute ventilation; VCO_2_, carbon dioxide production; VO_2_, oxygen consumption; RER, respiratory exchange ratio; HR, heart rate; SBP, systolic blood pressure; DBP, diastolic blood pressure; AT, anaerobic threshold; OUES, oxygen uptake efficiency slope; A-VO_2_Diff, arteriovenous oxygen difference.

Resting myocardial strain and myocardial work analysis were completed for all patients. Tables 3, 4 displayed the results for ESE variables in the overall population and each patient group. No significant differences in most baseline and low-load echocardiography parameters, including all myocardial work parameters, were observed between the groups. Compared with the normal group, the exercise intolerant group had a lower LVEF, mitral valve (MV) average S’, and LVGLS at rest but a higher EDV/E/e’ at low load. However, there was no significant difference between the two groups after the EDV/E/e’ normalization by BSA. A lower proportion of patients with exercise intolerance completed the exercise (achieved 85% of the age-predicted maximum heart rate) compared with that in the normal group. Also, the exercise intolerant group had lower SBP, SVi, and MV average S’ values and higher MV average E/e’ and EDVi/E/e’ at peak compared with the normal group.

**Table 3:** Baseline echocardiographic characteristics.

Variables	Total (*n* = 90)	Normal group (*n* = 36)	Exercise intolerant group (*n* = 54)	*P* value
Baseline				
LVEDV, ml	97.56 ± 20.68	92.71 ± 20.62	100.81 ± 20.27	0.068
LVEDVi, ml/m^2^	53.20 ± 8.91	52.14 ± 8.90	53.91 ± 8.92	0.367
LVESV, ml	30.46 ± 11.27	27.30 ± 9.41	32.57 ± 11.98	0.029
LVMi, g/m^2^	92.73 ± 21.37	90.48 ± 21.17	94.23 ± 21.56	0.418
RWT	0.40 ± 0.05	0.41 ± 0.05	0.41 ± 0.05	0.504
SV, ml	73.61 ± 14.09	72.65 ± 13.03	74.06 ± 15.11	0.602
SVi, ml/m^2^	40.39 ± 6.50	41.30 ± 6.28	39.78 ± 6.63	0.275
CO, L	5.02 ± 1.03	4.88 ± 0.86	5.11 ± 1.13	0.308
CI, L/m^2^	2.75 ± 0.48	2.78 ± 0.44	2.74 ± 0.51	0.667
LVEF,%	69.33 ± 6.09	70.91 ± 4.94	68.28 ± 6.59	0.045
MV Average S’, cm/s	9.14 ± 1.66	9.47 ± 1.75	8.64 ± 1.38	0.02
MV Septal e’, cm/s	7.73 ± 2.01	7.36 ± 1.90	7.98 ± 2.07	0.152
MV Average E/e’	8.36 ± 1.94	8.63 ± 1.76	8.18 ± 2.04	0.281
EDV/E/e’, ml	12.32 ± 4.45	11.31 ± 3.93	12.99 ± 4.68	0.078
EDVi/E/e’, ml/m^2^	6.76 ± 2.19	6.36 ± 2.05	7.02 ± 2.26	0.161
RV S’, cm/s	12.93 ± 1.86	12.71 ± 1.97	13.09 ± 1.79	0.352
TAPSE, cm	22.73 ± 2.16	22.65 ± 2.05	22.78 ± 2.24	0.779
RV FAC,%	48.31 ± 5.25	48.60 ± 5.34	48.12 ± 5.23	0.674
LV diastolic function				
Normal	74 (82%)	31 (91%)	43 (80%)	0.431
Abnormal	16 (18%)	5 (9%)	11 (20%)	
LV strain and myocardial work analysis				
LVGLS, %	−19.63 ± 2.14	−20.37 ± 1.44	−19.20 ± 2.38	0.009
GWI, mmHg%	1,868.94 ± 322.28	1,944.29 ± 252.77	1,820.11 ± 353.96	0.076
GCW, mmHg%	2,087.03 ± 336.09	2,165.46 ± 261.95	2,036.20 ± 369.91	0.076
GWW, mmHg%	97.11 ± 49.42	91.57 ± 47.47	100.70 ± 50.76	0.398
GWE, %	95 (94,96)	95 ± 2	95 (94,96)	0.223

EDVi, end-diastolic volume index; ESV, end-systolic volume; LVMi, left ventricle mass index; RWT, relative wall thickness; SVi, stroke volume index; CO, cardiac output; CI, cardiac index; LVEF, left ventricular ejection fraction; MV, mitral valve; TV, tricuspid valve; TAPSE, tricuspid annular plane systolic excursion; RV FAC, right ventricle fractional area change; LVGLS, left ventricular global longitudinal strain; GWI, global work index; GCW, global constructive work; GWW, global wasted work; GWE, global work efficiency.

**Table 4:** Exercise stress echocardiography characteristics.

Variables	Total (*n* = 90)	Normal group (*n* = 36)	Exercise intolerant group (*n* = 54)	*P* value
At low load				
HR beats/minute	94 ± 10	93 ± 11	95 ± 11	0.566
SBP, mmHg	147 ± 21	146 ± 23	147 ± 20	0.876
DBP, mmHg	81 ± 18	78 ± 18	83 ± 18	0.258
LV EDV, ml	101.44 ± 24.43	94.70 ± 25.34	106.16 ± 22.89	0.033
LV EDVi, ml/m^2^	54.04 ± 13.86	53.80 ± 13.00	54.20 ± 14.55	0.896
LVEF,%	75.27 ± 6.67	75.54 ± 5.50	75.07 ± 7.42	0.749
SV, ml	85.52 ± 17.87	83.31 ± 16.64	87.06 ± 18.69	0.343
SVi, ml/m^2^	46.79 ± 8.10	46.99 ± 7.56	46.65 ± 8.53	0.850
MV Average S’, cm/s	10.51 ± 1.99	10.21 ± 1.56	10.71 ± 2.24	0.256
MV Septal e’, cm/s	9.84 ± 2.50	10.22 ± 3.18	9.58 ± 1.89	0.247
MV Average E/e'	9.67 ± 2.42	9.92 ± 2.35	9.50 ± 2.47	0.436
EDV/E/e’, ml	11.10 ± 3.73	9.97 ± 3.10	11.90 ± 3.95	0.018
EDVi/E/e’, ml/m^2^	5.89 ± 1.97	5.67 ± 1.74	6.04 ± 2.12	0.391
At peak				
HR,% predicted at peak	78.00 (71.75–85)	80 (72.75–85)	75.78 ± 8.43	0.054
Reached the diagnostic endpoints	31 (34%)	14 (39%)	17 (31%)	<0.001
HR	137 ± 18	137 ± 18	138 ± 19	0.868
SBP, mmHg	179 ± 26	187 ± 26	174 ± 26	0.028
DBP, mmHg	87 ± 11	84 ± 10	87 ± 11	0.238
LVEDV, ml	101.30 ± 24.46	92.91 ± 23.31	107.36 ± 23.69	0.008
LVEDVi, ml/m^2^	55.12 ± 9.95	52.72 ± 10.03	56.85 ± 9.64	0.065
LVESV, ml	19.98 ± 10.15	16.97 ± 6.81	22.16 ± 11.59	0.022
LVEF,%	81.45 (79–84.83)	81.95 ± 4.11	81.00 (78.89–84.00)	0.327
SV, ml	94.07 ± 17.38	97.99 ± 16.62	91.38 ± 17.53	0.080
SVi, ml/m^2^	51.70 ± 8.23	55.76 ± 7.80	48.92 ± 7.37	<0.001
MV Average S’, cm/s	12.03 ± 2.12	12.43 ± 2.31	11.44 ± 1.66	0.037
MV Septal e’, cm/s	11.69 ± 3.14	11.50 ± 3.83	11.82 ± 2.58	0.650
MV Average E/e’	10.31 ± 2.92	9.65 ± 2.70	11.25 ± 2.99	0.017
EDV/E/e’, ml	10.72 ± 4.27	8.94 ± 3.89	11.98 ± 4.11	0.001
EDVi/E/e’, ml/m^2^	5.81 ± 2.06	5.04 ± 1.86	6.34 ± 2.04	0.005
Abnormal LV diastolic function	27 (30%)	10 (28%)	17 (31%)	<0.001

HR, heart rate; SBP, systolic blood pressure; DBP, diastolic blood pressure; EDVi, end-diastolic volume index; LVEF, left ventricular ejection fraction; SVi, stroke volume index; MV, mitral valve.

11 cases with normal left ventricular diastolic function at rest were reclassified as abnormal at peak. The exercise-intolerant group had a higher proportion of patients with abnormal left ventricular diastolic function. In the group with normal exercise tolerance, we did not detect segmental wall motion abnormalities at rest, even at peak. In contrast, in the exercise intolerant group, segmental wall motion abnormalities were detected in eight patients at rest [mean wall motion score index (WMSI) of 1.42], with no additional cases detected at peak. The mean WMSI of these eight patients was reduced (1.28) at peak.

Table 5 showed a comparison of exercise contractile reserve (*Δ*) between the two groups. Statistically significant differences in *Δ*SV, *Δ*SVi, *Δ*MV average E/e’, and flow reserve were observed between the two groups. The exercise intolerant group had a lower flow reserve, but the mean value (0.24) was higher than 0.20. Supplementary Table S3 reported the reproducibility analysis of echocardiographic parameters.

**Table 5:** Comparison of peak-resting echocardiographic parameters (*Δ*) between CCS patients with normal exercise tolerance and exercise intolerant.

Variables (*Δ*)	Total (*n* = 90)	Normal group (*n* = 36)	Exercise intolerant group (*n* = 54)	*P* value
*Δ*HR, beats/minute	66 ± 17	67 ± 17	66 ± 17	0.591
*Δ*SBP, mmHg	60 ± 28	67 ± 32	56 ± 24	0.084
*Δ*DBP, mmHg	14 ± 12	14 ± 12	13 ± 12	0.497
*Δ*LVEDV, ml	4.31 ± 18.79	1.61 ± 17.26	6.26 ± 19.78	0.275
*Δ*LVEDVi, ml/m^2^	2.11 ± 9.97	0.80 ± 9.49	3.05 ± 10.31	0.321
*Δ*LVESV, ml	−10.11 ± 6.19	−9.35 ± 6.24	−10.66 ± 6.16	0.351
*Δ*LVEF,%	11.17 ± 4.32	10.82 ± 4.14	11.42 ± 4.47	0.533
*Δ*SV, ml	20.31 ± 7.36	25.13 ± 6.32	17.00 ± 6.13	<0.001
*Δ*SVi, ml/m^2^	11.20 ± 4.03	14.31 ± 3.34	9.07 ± 2.94	<0.001
*Δ*MV Average S’	2.83 ± 1.83	2.89 ± 1.55	2.79 ± 2.01	0.809
*Δ*MV Septal e’	3.95 ± 3.09	4.18 ± 3.82	3.79 ± 2.48	0.578
*Δ*MV Average E/e’	1.85 ± 2.64	1.35 ± 2.65	2.57 ± 2.50	0.037
*Δ*EDV/E/e’	−1.74 ± 4.00	−2.14 ± 3.80	−1.46 ± 4.16	0.451
*Δ*EDVi/E/e’	−1.34 ± 2.47	−1.24 ± 2.13	−0.73 ± 1.96	0.280
Flow reserve	0.28 ± 0.10	0.35 ± 0.09	0.24 ± 0.09	<0.001

HR, heart rate; SBP, systolic blood pressure; DBP, diastolic blood pressure; EDVi, end-diastolic volume index; ESV, end-systolic volume; LVEF, left ventricular ejection fraction; SVi, stroke volume index; MV, mitral valve.

### Determinants of exercise capacity in patients with CCS

Supplementary Figures S1, S2 present the linear correlation analyses between echocardiographic parameters and peak oxygen consumption, as well as the %pred VO_2_ at peak. The correlation between flow reserve and peak oxygen consumption was the strongest, with a coefficient of 0.364 [95% confidence interval (CI), 0.156–0.528]. Meanwhile, the correlation between *Δ*SVi and%pred VO_2_ at the peak was the strongest, with a correlation coefficient of 0.607 (95%CI, 0.454–0.726).

**Figure 1:**
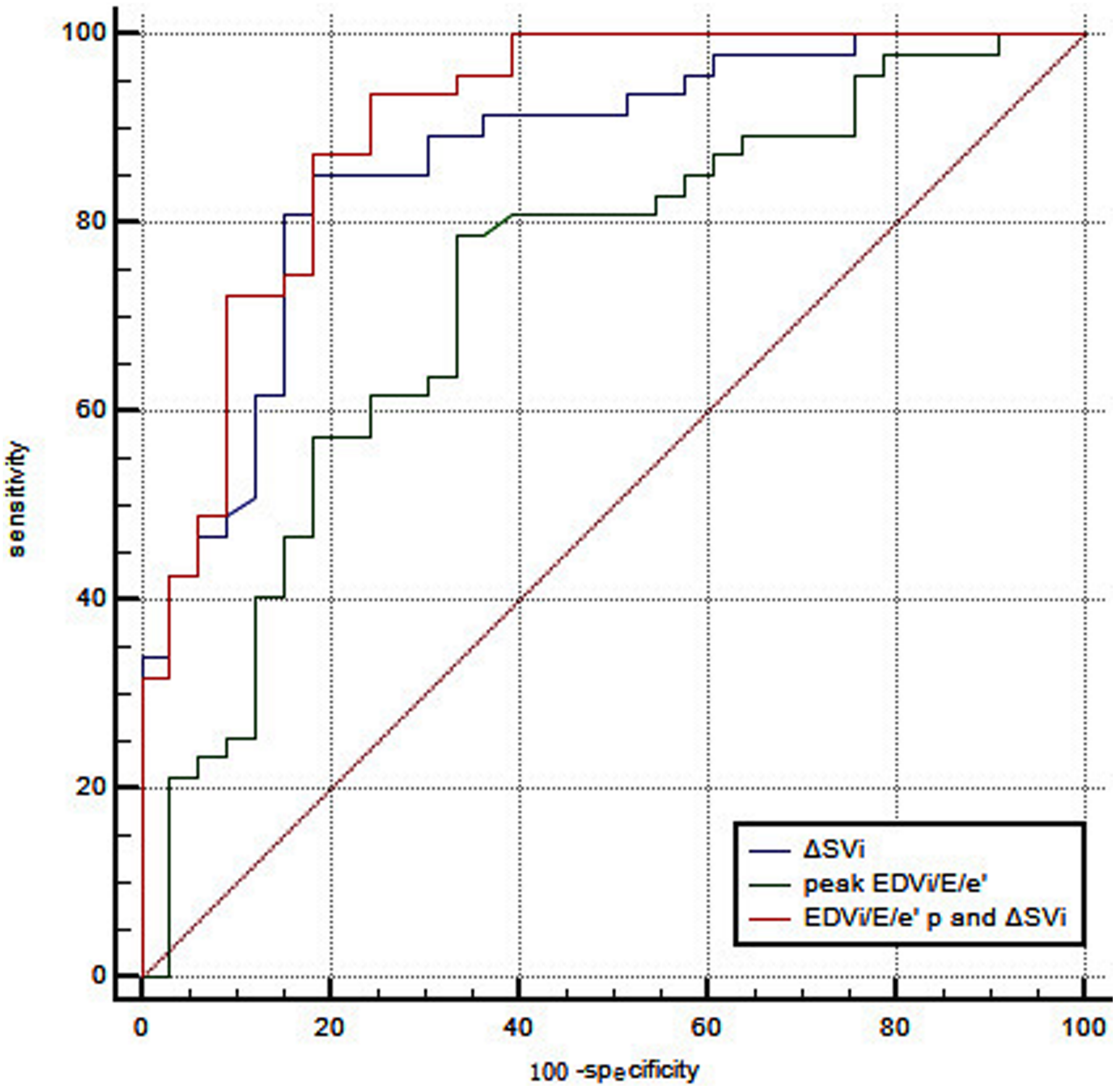
ROC curve analysis of peak EDVi/E/e’ combined with *Δ*SVi in predicting exercise intolerance in patients with CCS. With a cutoff value for peak EDVi/E/e’ of 8.64 ml/m^2^, the sensitivity and specificity were 78.7% and 66.7%, respectively, the area under the ROC curve of 0.734 (95% CI, 0.623–0.827). With a cutoff value for *Δ*SVi of 12.17 ml/m^2^, the sensitivity and specificity were 86.3% and 82.9%, respectively, the area under the ROC curve of 0.868 (95% CI, 0.774–0.933). The combination of these two parameters with a C-statistic of 0.906 (95%CI, 0.820−0.960) showed incremental predictive value in patients with CCS over peak EDVi/E/e’ alone (0.906 vs. 0.734, *P* = 0.001), but not *Δ*SVi (0.906 vs. 0.868, *P* = 0.158).

No independent correlation was found between clinical parameters, including age, gender, smoking history, previous PCI, and exercise intolerance in patients with CCS after multivariate stepwise logistic regression analysis. Of all the echocardiographic parameters, only the peak EDVi/E/e’ and *Δ*SVi independently correlated with exercise intolerance in patients with CCS (Table 6). With a cutoff value for peak EDVi/E/e’ of 8.64 ml/m^2^, the sensitivity and specificity were 78.7% and 66.7%, respectively, the area under the ROC curve of 0.734 (95% CI, 0.623–0.827) for the prediction of exercise intolerance in CCS patients. With a cutoff value for *Δ*SVi of 12.17 ml/m^2^, the sensitivity and specificity were 86.3% and 82.9%, respectively, the area under the ROC curve of 0.868 (95% CI, 0.774–0.933) for the prediction of exercise intolerance in CCS patients. The combination of these two parameters with a C-statistic of 0.906 (95%CI, 0.820–0.960) showed incremental predictive value in patients with CCS over peak EDVi/E/e’ alone (0.906 vs. 0.734, *P* = 0.001), but not *Δ*SVi (0.906 vs. 0.868, *P* = 0.158) (Figure 1). Supplementary Figure S3 summarizes the ESE-derived parameters used in the functional model to predict%pred VO_2_ at peak in patients with CCS.

**Table 6:** Univariate and multivariate logistic regression analysis for predictors of exercise intolerance in patients with CCS.

Variables	Univariable (OR, 95%CI)	*P* value	Multivariable (OR,95%CI)	*P* value
Age	0.92 (0.88–0.97)	0.001		
Gender	0.22 (0.09–0.56)	0.001		
Smoking history	3.02 (1.07–8.56)	0.038		
Previous PCI	4.95 (1.03–23.71)	0.045		
LVEFr	0.92 (0.84–1.0)	0.050		
S'r	1.39 (1.04–1.85)	0.024		
LVGLS	1.24 (0.98–1.57)	0.068		
SVip	0.89 (0.83–0.95)	<0.001		
S'p	1.28 (1.01–1.61)	0.042		
MV Average E/e'p	0.82 (0.69–0.97)	0.019		
EDVi/E/e'p	1.23 (1.07–1.41)	0.003	1.27 (1.06–1.52)	0.008
Flow Reserve	0.86 (0.80–0.92)	<0.001		
*Δ*SVi	0.57 (0.45–0.72)	<0.001	0.57 (0.44–0.74)	<0.001
*Δ*MV Average E/e’	0.82 (0.68–1.00)	0.045		
Abnormal LV diastolic function at peak	0.92 (0.35–2.40)	0.866		

PCI, percutaneous coronary intervention; LVEF, left ventricular ejection fraction; LVGLS, left ventricular global longitudinal strain; SVi, stroke volume index; MV, mitral valve; EDVi, end-diastolic volume index.

## Discussion

**Figure 2:**
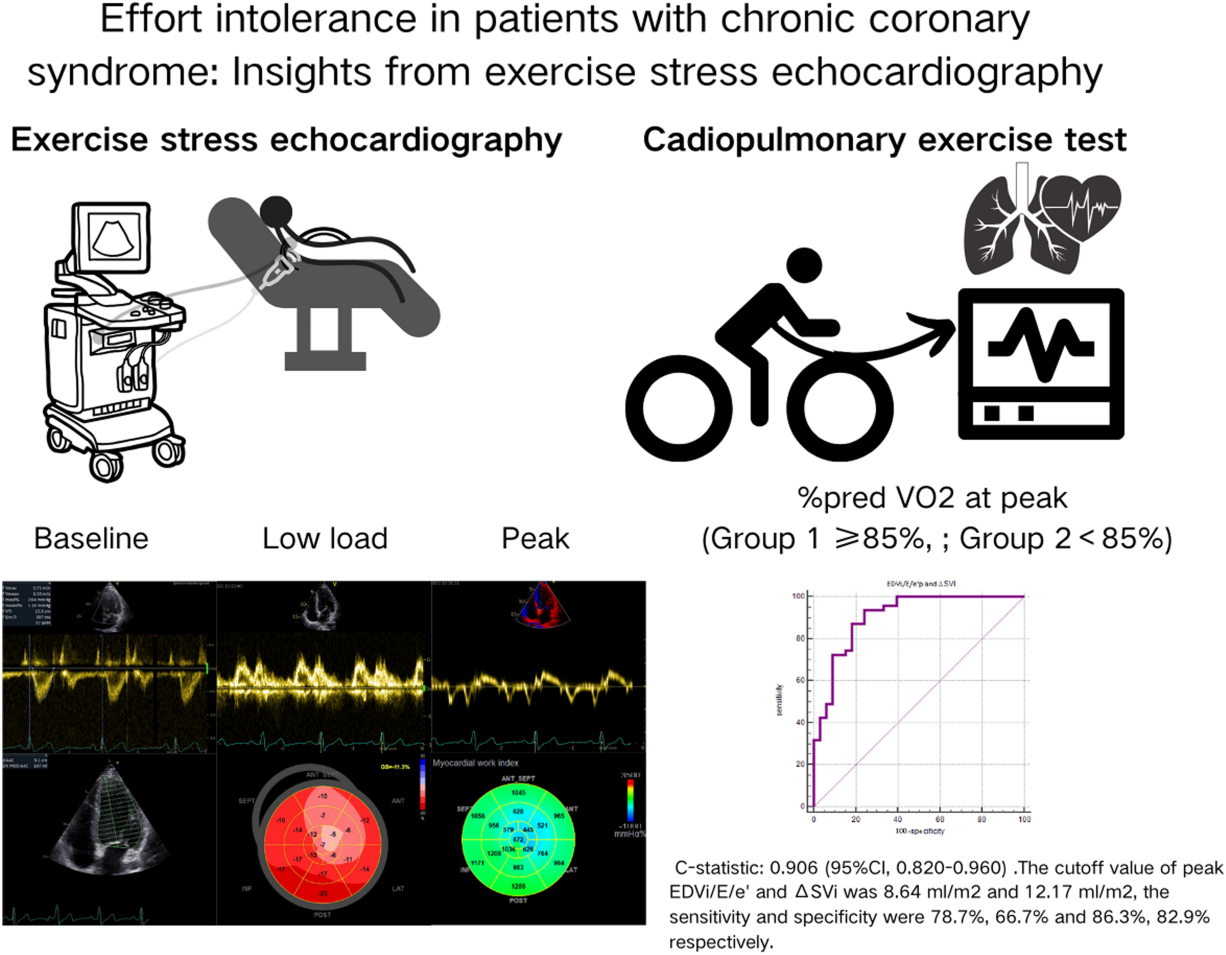
The main procedures and findings of our study. All patients underwent CPET and ESE. We analyzed the exercise stress echocardiography images in detail, including the traditional two-dimensional parameters, myocardial strain and myocardial work parameters. Finally, we found that only the peak EDVi/E/e’ and *Δ*SVi independently correlated with exercise intolerance in patients with CCS.

The main findings of the present study are that peak EDVi/E/e’ and *Δ*SVi were identified as independent risk factors for exercise intolerance in patients with CCS, while all resting echocardiographic parameters, including myocardial work parameters and flow reserve, were not (Figure 2). Our study was a relatively rare study that combined ESE and CPET to analyze the mechanism of exercise intolerance in patients with CCS. Numerous previous studies have focused on investigating the possible causes of exercise intolerance in patients with heart failure, with the results showing that patients with heart failure with reduced ejection fraction (HFrEF) presented with reduced peak VO_2_, mainly as a result of chronotropic incompetence and inadequate SV increase. In contrast, patients with heart failure with preserved ejection fraction (HFpEF) developed multiple alterations of the cardiovascular function involving both central and peripheral components (13, 17–19). It is not uncommon for patients with CCS to have HFpEF or HFrEF, indicating the possibility of overlapping mechanisms for their exercise intolerance. Therefore, We hypothesized that CPET combined with ESE could analyze the mechanism of exercise intolerance in patients with CCS and ultimately found that *Δ*SVi and peak EDVi/E/e’ were independent risk factors. Rozenbaum et al. also found that a combined ESE with CPET, measuring stroke volume and AVO_2_ difference throughout the effort, may help diagnose significant coronary artery disease (20). However, the Smarz team (21, 22) analyzed the mechanism of exercise intolerance in patients after PCI for acute myocardial infarction and obtained different results, showing that exercise intolerance was related to slower heart rate response and decreased peak peripheral blood oxygen uptake (A-VO2Diff) during exercise, but not to stroke volume. Smarz's study involved fewer echocardiographic parameters and did not include parameters such as systolic function reserve, which reflected the hemodynamic changes during exercise. The conclusions of this study were only applicable to patients with acute myocardial infarction with LVEF ≥ 40% after PCI. Our study focuses on CCS patients, the most common type among CAD but is often overlooked due to its subtle symptoms and the limited information provided by resting echocardiography.

Several causes of exercise intolerance, leading to low%pred VO_2_, have been described, including Fick and non-Fick variables. We excluded patients with non-Fick variables that led to exercise termination, including pulmonary and peripheral nervous system diseases, peripheral arterial or vein pathologies, or bone and joint abnormalities. The Fick variables mainly comprise three significant elements: stroke volume, heart rate, and A-VO_2_Diff (23). Patients with CCS experience a series of ischemic cascade reactions triggered by coronary artery lesions (narrowing, spasm, or microvascular disease), which lead to reduced local blood flow perfusion, imbalance between myocardial oxygen supply and demand, decreased local myocardial contractility and echocardiographic manifestations of resting or stress-induced segmental wall motion abnormalities or reduced left ventricular systolic reserve. Subsequently, left ventricular end-diastolic pressure increases, followed by a decrease in diastolic and global systolic functions, resulting in changes in the electrocardiogram, chest pain, exercise intolerance, and other clinical manifestations (24–26). In addition to segmental wall motion abnormalities, echocardiography also detects abnormal results for diastolic function parameters in patients with CCS, such as E/e’, EDV/E/e,’ as well as systolic function parameters, including LVEF, S,’ LVGLS, myocardial work parameters and SV. We observed differences in the mitral valve average E/e’ at peak, S’ at rest and peak, LVEF, and LVGLS at rest between the groups of CCS patients with and without exercise intolerance. However, on multivariate analysis, only the peak EDVi/E/e’ and *Δ*SVi, reflecting left ventricular compliance and contractile reserve, respectively, showed independent correlations with exercise intolerance in patients with CCS.

EDV/E/e’ was originally proposed by Shimiaie et al., reflecting the relationship between left ventricular filling pressure and volume rather than just left ventricular end-diastolic pressure or stroke volume alone (12). Changes in this ratio better represent the inability of the LV to increase its size, even with high filling pressure, suggesting a decrease in left ventricular compliance. The peak EDV/E/e’ in patients with CCS was significantly lower than at rest, regardless of exercise tolerance. Considering the differences in BSA between the two groups of patients in our study cohort, we used BSA to standardize EDV/E/e’ to obtain EDVi/E/e’. Our study showed that higher peak EDVi/E/e’ values were associated with exercise intolerance in patients with CCS, inconsistent with previous studies in HFpEF (12). We analyze the possible reasons as follows: firstly, there is a difference in age between the normal exercise tolerance and exercise intolerance groups in our study, and E/e’ gradually increases with age, which may lead to a higher EDVi/E/e’ value in the exercise intolerance group. Although we adjusted age factors in multivariate analysis, their effect on E/e’ could not be corrected. Secondly, coronary circulation dysfunction may directly lead to a gradual increase in left ventricular size, which may be another important reason. Nevertheless, these findings should be considered hypothesis-generating and tested with prospective invasive methods. Our study also showed that its sensitivity and specificity in predicting exercise intolerance are not high, and SV combined with EDVi/E/e’ has an incremental value compared to EDVi/E/e’ alone in predicting exercise intolerance in patients with CCS.

Although many previous studies have proven the value of LV GLS and myocardial work parameters in diagnosing severe CAD and predicting future cardiovascular events (27–31). Our study focused on analyzing risk factors associated with exercise intolerance in patients with CCS rather than predicting severe CAD, and only 19 patients had a single vessel stenosis rate ≥50% on CCTA. Myocardial ischemic injury may only be one factor leading to exercise intolerance in CCS patients; when left ventricular systolic function is preserved, diastolic dysfunction and reduced left ventricular systolic reserve become more critical. Among the three significant Fick variables, our study found that only *Δ*SVi independently correlated with reduced exercise capacity in patients with CCS, while heart rate response and A-VO_2_Diff did not. One possible reason is that the included cases had relatively mild coronary artery stenosis, with only 21% having single-vessel stenosis >50%. Another reason could be that our study did not strictly control the use of beta-blockers by patients on the day of examination, making the study situation closer to clinical reality. Despite this, we still observed differences in the percentages of patients who reached the predicted percentage of maximum heart rate during ESE between the normal group and exercise intolerant group. Additionally, we conducted CPET and ESE using different devices due to equipment limitations. AVO_2_diff, a non-invasive parameter derived from CPET-ESE, may exhibit calculation bias in our study.

### Clinical implications

CCS, the most common and widely distributed type in CAD, requires long-term follow-up and management. Due to its subtle symptoms, patients may not be aware of their condition, leading to frequent neglect in clinical practice. Exercise intolerance in CCS patients poses a risk of progressing to heart failure and ACS. Conventional resting echocardiography provides minimal information and often reveals no abnormalities. The combined application of CPET and ESE allows for assessing exercise capacity and further evaluating left ventricular diastolic and systolic function and exercise systolic reserve. This combination also facilitates the investigation of the specific causes of exercise intolerance in patients with CCS, supporting the administration of targeted and timely treatment to improve patients’ exercise capacity and reduce the occurrence of adverse cardiovascular outcomes.

CPET acquisition can be completed while performing ESE in a semi-recumbent position for qualified cardiac centres. We recommend that patients with CCS who had exercise ability in these cardiac centres should complete these two tests. Judging from the results of our study, it seems that patients with mild coronary stenosis have more significant benefits from these two examinations. Therefore, for patients with clinically suspected CCS but no significant coronary stenosis on CCTA, CPET and ESE examinations help detect exercise tolerance and aetiology analysis. Our study revealed that peak EDVi/E/e’ and *Δ*SVi were identified as independent risk factors for exercise intolerance in patients with CCS. In clinical practice, targeted exercise training should be provided for these patients with exercise intolerance, and they were reexamined after some time training to observe whether peak EDVi/E/e’ and *Δ*SVi had improved while exercise capacity was recovered.

### Limitations

Various limitations of the current work should be noted. This was a single-centre study with a relatively small sample size. Further multicenter studies with large samples are needed to confirm our preliminary findings. Patient selection was also biased, excluding those unwilling to undergo two exercise tests. This directly led to fewer patients with LVEF of 40%–50%, so we could not conduct further subgroup analysis based on LVEF. In our study, only 19 patients (21%) had single-vessel stenosis >50%, and 5 patients in the group with normal exercise capacity. The small number of positive cases was insufficient to support further stratified analyses for plaque burden and the coronary artery affected by CAD. Our future research will focus on the impact of plaque burden on exercise capacity and prognosis of patients with CCS, but it was not the focus of this study. In addition, ESE was more challenging in obtaining coronary flow velocity reserve (CFVR) due to the hyperventilation and motion of patients during peak exercise. So, we gave up measuring CFVR, which may be related to the exercise intolerance of CCS patients. Due to equipment limitations, the separated administration of CPET and ESE could result in bias in the calculation of AVO_2_diff. Our study also did not specify the types of CCS. The subsequent analysis of any differences in the mechanisms of exercise intolerance among different types of CCS will be a focus of our future research. Previous studies have shown that the increased left atrial volume during exercise in CCS patients is related to decreased left ventricular systolic reserve (32). Our study did not analyze whether changes in left atrial structure and function are related to exercise intolerance in CCS patients, and this topic warrants investigation in the future. Although our study excluded patients with poor acoustic windows, challenges persist in measuring echocardiographic parameters during exercise due to gas interference.

## Conclusions

CPET is a valuable tool for evaluating the exercise capacity of CCS patients, and its combination with ESE further supports the analysis of the reasons for exercise intolerance. Exercise intolerance in CCS patients is complex and may result from the combined effects of multiple mechanisms. Our study showed that only peak EDVi/E/e’ and *Δ*SVi were independent predictive factors for exercise intolerance in the included CCS patients. Therefore, in clinical practice, exercise stress echocardiography may be an essential addition to routine resting echocardiography for CCS patients.

## Data Availability

The original contributions presented in the study are included in the article/Supplementary Material, further inquiries can be directed to the corresponding author.

## Supplementary material

Supplementary Figure 1 The linear correlation between echocardiographic parameters and peak oxygen consumption, as well as%pred VO_2_ at peak. **(A)** The linear correlation between Sr and peak VO_2_, *r* = 0.262 (95%CI, 0.055–0.448), *P* value = 0.014; **(B)** the linear correlation between Sp and peak VO_2_, *r* = 0.311(95%CI, 0.100–0.500), *P* value = 0.005; **(C)** the linear correlation between *Δ*SVi and peak VO_2_, *r* = 0.356 (95%CI, 0.156–0.528), *P* value = 0.0008; **(D)** the linear correlation between EDVi/E/e’ and peak VO_2_, *r* = 0.273 (95%CI, 0.057–0.465), *P* value = 0.014; **(E)** the linear correlation between flow reserve and peak VO_2_, *r* = 0.364 (95%CI, 0.165–0.535), *P* value = 0.0006; **(F)** the linear correlation between *Δ*SVi and%pred VO_2_ at peak, *r* = 0.607 (95%CI, 0.404–0.720), *P* value < 0.0001.

Supplementary Figure 2 The linear correlation between echocardiographic parameters and%pred VO_2_ at peak. **(A)** The linear correlation between LVESVr and%pred VO_2_ at peak, *r* = −0.218 (95%CI, −0.008 to −0.410), *P* value = 0.043; **(B)** the linear correlation between LVEFr and%pred VO_2_ at peak_,_ *r* = 0.243(95%CI, 0.034–0.431), *P* value = 0.023; **(C)** the linear correlation between LVGLS and%pred VO_2_ at peak, *r* = −0.346 (95%CI, −0.146 to −0.519), *P* value = 0.001; **(D)** the linear correlation between SVip and%pred VO_2_ at peak, *r* = 0.512 (95%CI, 0.336–0.653), *P* value < 0.0001; **(E)** the linear correlation between EDVi/E/e’ and%pred VO_2_ at peak, *r* = −0.232 (95%CI, −0.013 to −0.430), *P* value = 0.039; **(F)** the linear correlation between flow reserve and%pred VO_2_ at peak, *r* = 0.451 (95%CI, 0.264–0.605), *P* value < 0.0001.

Supplementary Figure 3 Summarizes the ESE-derived parameters used in the functional model to predict%pred VO_2_ at peak in patients with CCS.

## References

[1] Townsend N Nichols M Scarborough P Rayner M. Cardiovascular disease in Europe–epidemiological update 2015. Eur Heart J. (2015) 36:2696–705. 10.1093/eurheartj/ehv428 26306399

[2] Knuuti J Wijns W Saraste A Capodanno D Barbato E Funck-Brentano C 2019 ESC guidelines for the diagnosis and management of chronic coronary syndromes. Eur Heart J. (2020) 41:407–77. 10.1093/eurheartj/ehz425 31504439

[3] Kaminsky LA Myers J Arena R. Determining cardiorespiratory fitness with precision: compendium of findings from the FRIEND registry. Prog Cardiovasc Dis. (2019) 62:76–82. 10.1016/j.pcad.2018.10.003 30385268

[4] Kiviniemi AM Lepojarvi S Kentta TV Junttila MJ Perkiomaki JS Piira OP Exercise capacity and heart rate responses to exercise as predictors of short-term outcome among patients with stable coronary artery disease. Am J Cardiol. (2015) 116:1495–501. 10.1016/j.amjcard.2015.08.014 26381535

[5] Hung RK Al-Mallah MH McEvoy JW Whelton SP Blumenthal RS Nasir K Prognostic value of exercise capacity in patients with coronary artery disease: the FIT (henry ford ExercIse testing) project. Mayo Clin Proc. (2014) 89:1644–54. 10.1016/j.mayocp.2014.07.011 25440889

[6] Guazzi M Bandera F Ozemek C Systrom D Arena R. Cardiopulmonary exercise testing J Am Coll Cardiol. (2017) 70:1618–36. 10.1016/j.jacc.2017.08.012 28935040

[7] Guazzi M Arena R Halle M Piepoli MF Myers J Lavie CJ. 2016 focused update: clinical recommendations for cardiopulmonary exercise testing data assessment in specific patient populations. Circulation. (2016) 133:e694–711. 10.1161/CIR.0000000000000406 27143685

[8] Santoro C Sorrentino R Esposito R Lembo M Capone V Rozza F Cardiopulmonary exercise testing and echocardiographic exam: an useful interaction. Cardiovasc Ultrasound. (2019) 17:29. 10.1186/s12947-019-0180-0 31796047 PMC6892222

[9] Lang RM Badano LP Mor-Avi V Afilalo J Armstrong A Ernande L Recommendations for cardiac chamber quantification by echocardiography in adults: an update from the American society of echocardiography and the European association of cardiovascular imaging. J Am Soc Echocardiogr. (2015) 28:1–39.e14. 10.1016/j.echo.2014.10.003 25559473

[10] Pellikka PA Arruda-Olson A Chaudhry FA Chen MH Marshall JE Porter TR Guidelines for performance, interpretation, and application of stress echocardiography in ischemic heart disease: from the American society of echocardiography. J Am Soc Echocardiogr. (2020) 33:1–41.e8. 10.1016/j.echo.2019.07.001 31740370

[11] Nagueh SF Smiseth OA Appleton CP Byrd BF 3rd Dokainish H Edvardsen T Recommendations for the evaluation of left ventricular diastolic function by echocardiography: an update from the American society of echocardiography and the European association of cardiovascular imaging. J Am Soc Echocardiogr. (2016) 29:277–314. 10.1016/j.echo.2016.01.011 27037982

[12] Shimiaie J Sherez J Aviram G Megidish R Viskin S Halkin A Determinants of effort intolerance in patients with heart failure. JACC Heart Fail. (2015) 3:803–14. 10.1016/j.jchf.2015.05.010 26449998

[13] Pugliese NR De Biase N Conte L Gargani L Mazzola M Fabiani I Cardiac reserve and exercise capacity: insights from combined cardiopulmonary and exercise echocardiography stress testing. J Am Soc Echocardiogr. (2021) 34:38–50. 10.1016/j.echo.2020.08.015 33036818

[14] Bugge-Asperheim B Kiil F. Preload, contractility, and afterload as determinants of stroke volume during elevation of aortic blood pressure in dogs. Cardiovasc Res. (1973) 7:528–41. 10.1093/cvr/7.4.528 4721686

[15] Guazzi M Adams V Conraads V Halle M Mezzani A Vanhees L EACPR/AHA scientific statement. Clinical recommendations for cardiopulmonary exercise testing data assessment in specific patient populations. Circulation. (2012) 126:2261–74. 10.1161/CIR.0b013e31826fb946 22952317 PMC4777325

[16] Cardiovascular Branch of Chinese Medical Association, Professional Committee of Cardiopulmonary Prevention and Rehabilitation of China Rehabilitation Medicine Association, Editorial Committee of Chinese Journal of Cardiovascular Disease. Chinese expert consensus on standardized clinical application of cardiopulmonary exercise testing. J Chin Cardiovasc Dis. (2022) 50:973–86. 10.3760/cma.j.cn112148-20220316-00180

[17] Obokata M Olson TP Reddy YNV Melenovsky V Kane GC Borlaug BA. Haemodynamics, dyspnoea, and pulmonary reserve in heart failure with preserved ejection fraction. Eur Heart J. (2018) 39:2810–21. 10.1093/eurheartj/ehy268 29788047 PMC6658816

[18] Pugliese NR Fabiani I Santini C Rovai I Pedrinelli R Natali A Value of combined cardiopulmonary and echocardiography stress test to characterize the haemodynamic and metabolic responses of patients with heart failure and mid-range ejection fraction. Eur Heart J Cardiovasc Imaging. (2019) 20:828–36. 10.1093/ehjci/jez014 30753369

[19] Pugliese NR Mazzola M Fabiani I Gargani L De Biase N Pedrinelli R Haemodynamic and metabolic phenotyping of hypertensive patients with and without heart failure by combining cardiopulmonary and echocardiographic stress test. Eur J Heart Fail. (2020) 22:458–68. 10.1002/ejhf.1739 31950651

[20] Rozenbaum Z Khouri S Aviram G Gura Y Sherez J Man A Discriminating circulatory problems from deconditioning. Chest. (2017) 151:431–40. 10.1016/j.chest.2016.09.027 27742182

[21] Smarz K Jaxa-Chamiec T Zaborska B Tysarowski M Budaj A. Combined use of stress echocardiography and cardiopulmonary exercise testing to assess exercise intolerance in patients treated for acute myocardial infarction. PLoS One. (2021) 16:e0255682. 10.1371/journal.pone.0255682 34351993 PMC8341484

[22] Smarz K Jaxa-Chamiec T Zaborska B Tysarowski M Budaj A. Mechanisms of exercise capacity improvement after cardiac rehabilitation following myocardial infarction assessed with combined stress echocardiography and cardiopulmonary exercise testing. J Clin Med. (2021) 10:4083. 10.3390/jcm10184083 34575194 PMC8471103

[23] Houstis NE Lewis GD. Causes of exercise intolerance in heart failure with preserved ejection fraction: searching for consensus. J Card Fail. (2014) 20:762–78. 10.1016/j.cardfail.2014.07.010 25108084

[24] Nesto RW Kowalchuk GJ. The ischemic cascade: temporal sequence of hemodynamic, electrocardiographic and symptomatic expressions of ischemia. Am J Cardiol. (1987) 59:23C–30C. 10.1016/0002-9149(87)90192-5 2950748

[25] Chinese Society of Echocardiography, Shallow Tissue and Vascular Group of Chinese Medical Association Ultrasound Medicine Branch, Ultrasound Medicine Professional Committee of China Medical Education Association. Clinical practice guidelines for stress echocardiography in chronic coronary artery syndrome (2023 edition). Chin J Ultrason. (2023) 32:921–45. 10.3760/cma.j.cn131148-20230511-00267

[26] Gaibazzi N Ciampi Q Cortigiani L Wierzbowska-Drabik K Zagatina A Djordjevic-Dikic A Multiple phenotypes of chronic coronary syndromes identified by ABCDE stress echocardiography. J Am Soc Echocardiogr. (2024) 37:477–85. 10.1016/j.echo.2023.12.003 38092306

[27] Brainin P Hoffmann S Fritz-Hansen T Olsen FJ Jensen JS Biering-Sorensen T. Usefulness of postsystolic shortening to diagnose coronary artery disease and predict future cardiovascular events in stable angina pectoris. J Am Soc Echocardiogr. (2018) 31:870–879.e3. 10.1016/j.echo.2018.05.007 30077186

[28] Pastore MC Mandoli GE Contorni F Cavigli L Focardi M D'Ascenzi F Speckle tracking echocardiography: early predictor of diagnosis and prognosis in coronary artery disease. Biomed Res Int. (2021) 2021:1. 10.1155/2021/6685378 33623788 PMC7875622

[29] Borrie A Goggin C Ershad S Robinson W Sasse A. Noninvasive myocardial work index: characterizing the normal and ischemic response to exercise. J Am Soc Echocardiogr. (2020) 33:1191–200. 10.1016/j.echo.2020.05.003 32651126

[30] Edwards NFA Scalia GM Sabapathy S Anderson B Chamberlain R Khandheria BK Resting global myocardial work can improve interpretation of exercise stress echocardiography. Int J Cardiovasc Imaging. (2021) 37:2409–17. 10.1007/s10554-021-02216-0 33721155

[31] Gaibazzi N Bergamaschi L Pizzi C Tuttolomondo D. Resting global longitudinal strain and stress echocardiography to detect coronary artery disease burden. Eur Heart J Cardiovasc Imaging. (2023) 24:e86–8. 10.1093/ehjci/jead046 36935564

[32] Morrone D Arbucci R Wierzbowska-Drabik K Ciampi Q Peteiro J Agoston G Feasibility and functional correlates of left atrial volume changes during stress echocardiography in chronic coronary syndromes. Int J Cardiovasc Imaging. (2021) 37:953–64. 10.1007/s10554-020-02071-5 33057991

