# Supplementary figures and images for: Exercise intolerance in patients with chronic coronary syndrome: insights from exercise stress echocardiography
Source: Front Cardiovasc Med. 2024 Nov 28;11:1442263. doi: 10.3389/fcvm.2024.1442263 (PMC11634879; doi:10.3389/fcvm.2024.1442263)

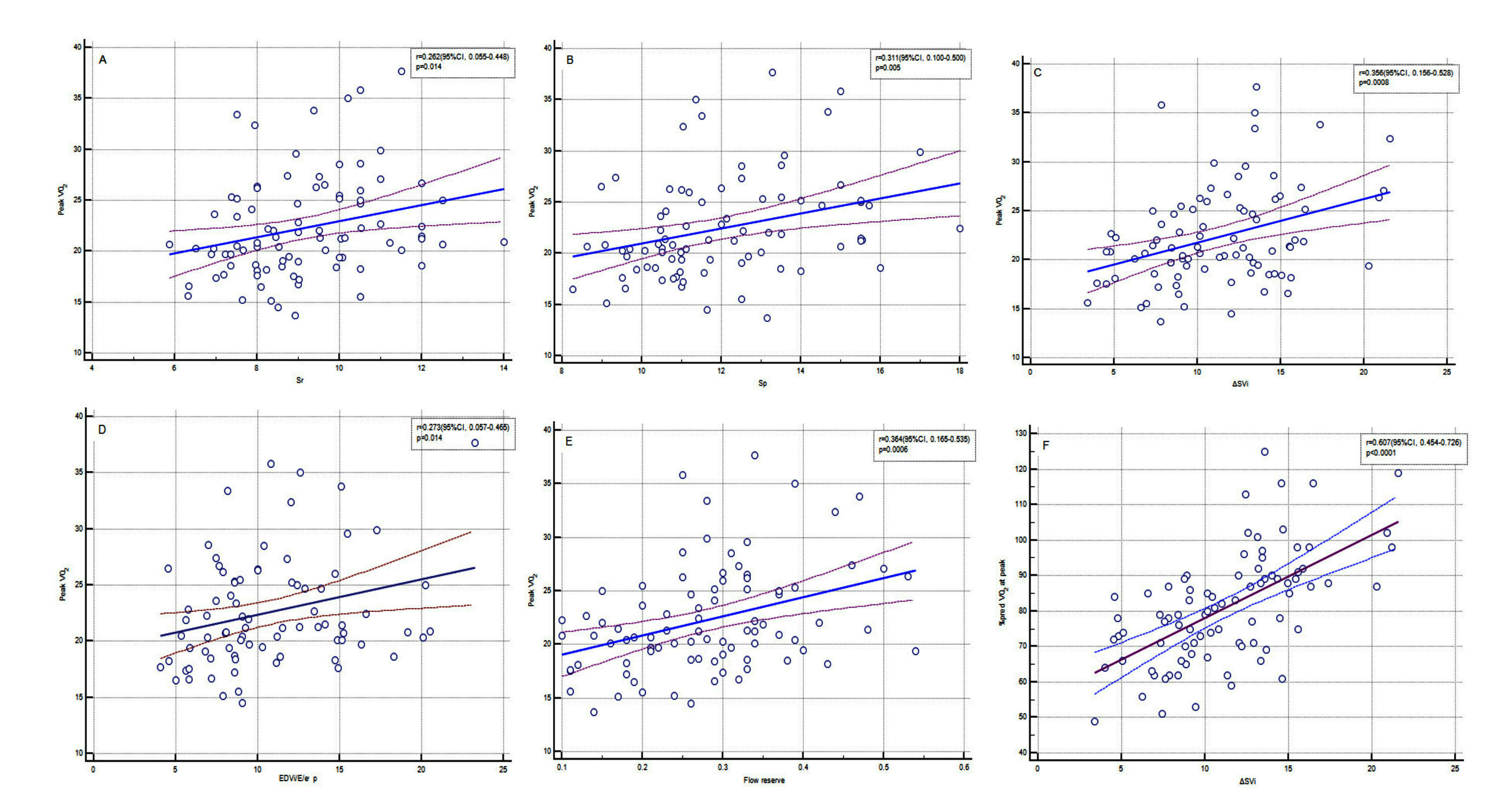

Supplement: Supplementary Figure 1 — The linear correlation between echocardiographic parameters and peak oxygen consumption, as well as%pred VO2 at peak. (A) The linear correlation between Sr and peak VO2, r = 0.262 (95%CI, 0.055–0.448), P value = 0.014; (B) the linear correlation between Sp and peak VO2, r = 0.311(95%CI, 0.100–0.500), P value = 0.005; (C) the linear correlation between ΔSVi and peak VO2, r = 0.356 (95%CI, 0.156–0.528), P value = 0.0008; (D) the linear correlation between EDVi/E/e’ and peak VO2, r = 0.273 (95%CI, 0.057–0.465), P value = 0.014; (E) the linear correlation between flow reserve and peak VO2, r = 0.364 (95%CI, 0.165–0.535), P value = 0.0006; (F) the linear correlation between ΔSVi and%pred VO2 at peak, r = 0.607 (95%CI, 0.404–0.720), P value < 0.0001. [file Image1.tiff]

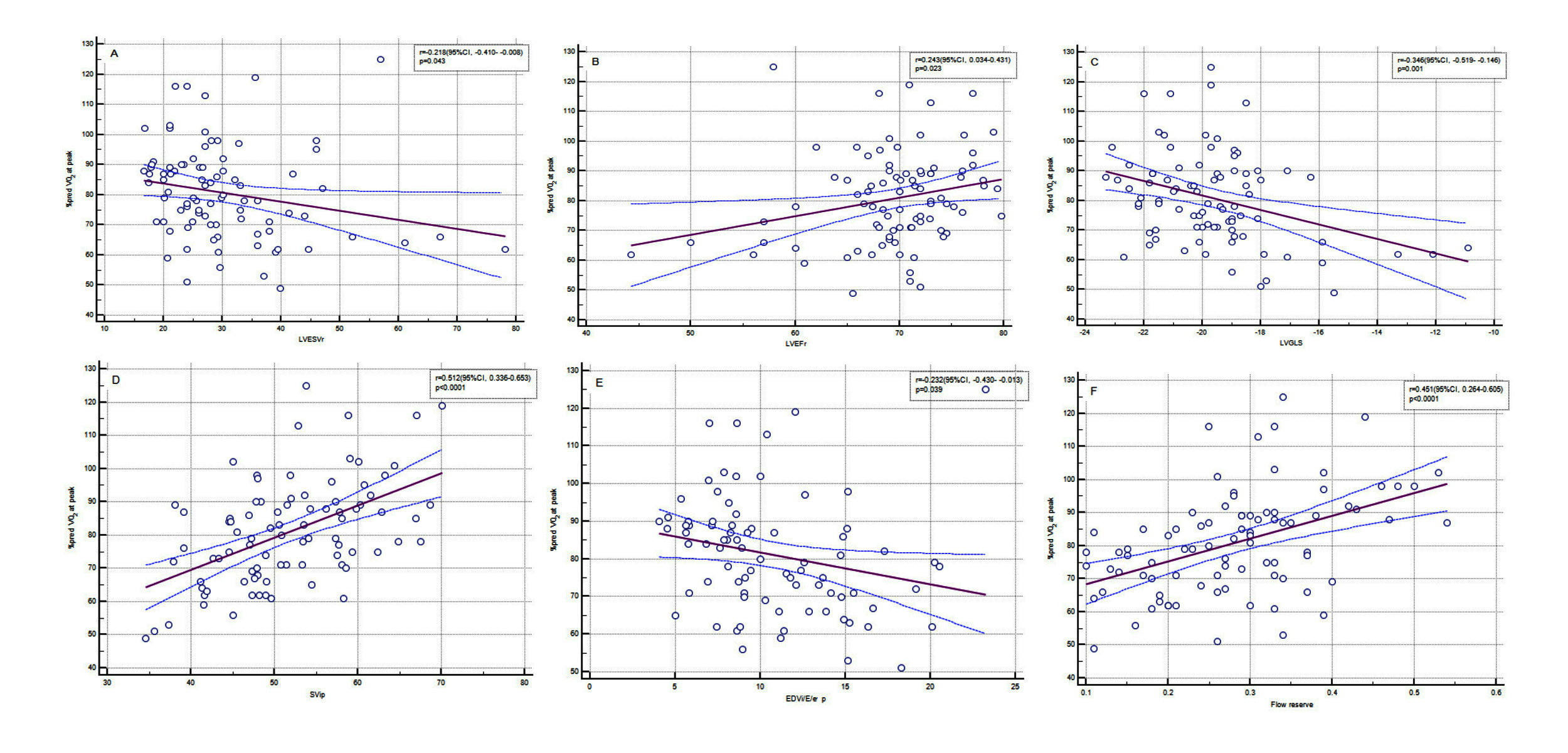

Supplement: Supplementary Figure 2 — The linear correlation between echocardiographic parameters and%pred VO2 at peak. (A) The linear correlation between LVESVr and%pred VO2 at peak, r = −0.218 (95%CI, −0.008 to −0.410), P value = 0.043; (B) the linear correlation between LVEFr and%pred VO2 at peak, r = 0.243(95%CI, 0.034–0.431), P value = 0.023; (C) the linear correlation between LVGLS and%pred VO2 at peak, r = −0.346 (95%CI, −0.146 to −0.519), P value = 0.001; (D) the linear correlation between SVip and%pred VO2 at peak, r = 0.512 (95%CI, 0.336–0.653), P value < 0.0001; (E) the linear correlation between EDVi/E/e’ and%pred VO2 at peak, r = −0.232 (95%CI, −0.013 to −0.430), P value = 0.039; (F) the linear correlation between flow reserve and%pred VO2 at peak, r = 0.451 (95%CI, 0.264–0.605), P value < 0.0001. [file Image2.tiff]

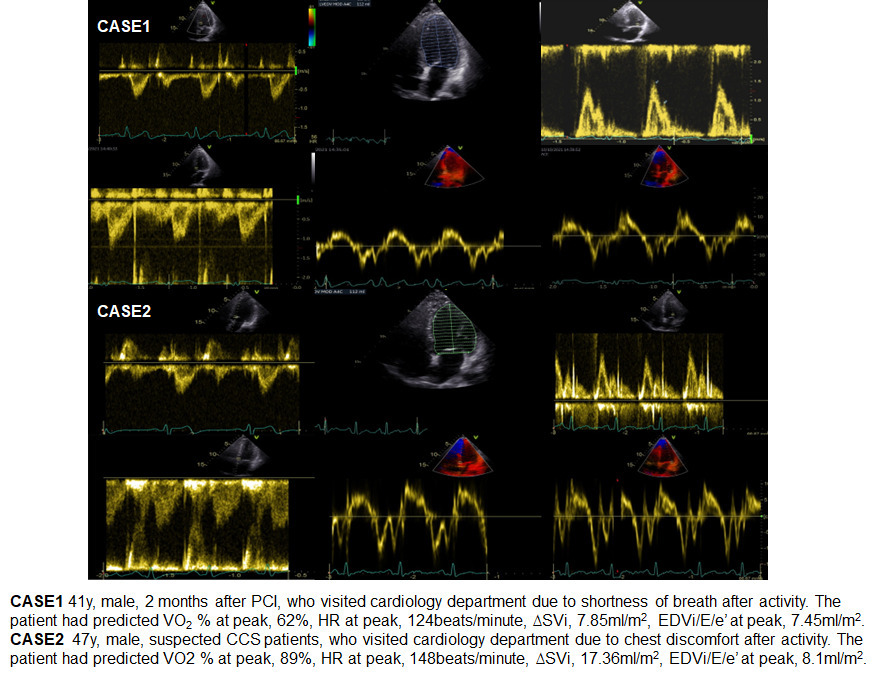

Supplement: Supplementary Figure 3 — Summarizes the ESE-derived parameters used in the functional model to predict%pred VO2 at peak in patients with CCS. [file Image3.tiff]
