# Supplementary material for: Exercise intolerance in patients with chronic coronary syndrome: insights from exercise stress echocardiography
Source: Front Cardiovasc Med. 2024 Nov 28;11:1442263. doi: 10.3389/fcvm.2024.1442263 (PMC11634879; doi:10.3389/fcvm.2024.1442263)
Supplement: Supplementary file 4 [file Table1.docx]

**Supplementary Table1 Baseline and ESE acquisition protocol**

|  | **Baseline** | **Low-load effort** | **peak effort** |
| --- | --- | --- | --- |
| Dynamic images | Two-dimensional cine loops from the apical four-, two-, and three-chamber views were acquired. Regional wall motion was assessed and graded using a 4-point scale, where 1 represented normal, and 4 represented dyskinetic motion in a 16-segment model and was expressed as a wall motion score index (WMSI) | | |
| echocardiographic parameters | LVEDV, LVEDVi, LVESV  LV EF, LVMi, RWT  SV, SVi, CO, CI  MV S', septal e', average E/e'  LVEDV/E/e', LVEDVi/E/e'  TV S', TAPSE, RV FAC | LVEDV, LVEDVi, LV EF  SV, SVi  MV S', septal e', average E/e'  LVEDV/E/e', LVEDVi/E/e' | LVEDV, LVEDVi, LVESV  LV EF, SV, SVi  MV S', septal e', average E/e'  LVEDV/E/e', LVEDVi/E/e' |

ESE, exercise stress echocardiography; LV EDV, left ventricular end-diastolic volume; EDVi, end-diastolic volume index; ESV, end-systolic volume; ESVi, end-systolic volume index; EF, ejection fraction; LVMi, left ventricle mass index; RWT, relative wall thickness; SV, stroke volume; SVi, stroke volume index; CO, cardiac output; CI, cardiac index; MV, mitral valve; TV, tricuspid valve; TAPSE, tricuspid annular plane systolic excursion; RV FAC, right ventricle fractional area change.

LV EDV and LV ESV were derived using the biplane Simpson's method. SV was measured by multiplying the LV outflow tract area at rest by the LV outflow tract velocity time integral measured by pulsed-wave Doppler during each activity level.

**Supplementary Table 2 Comparison of cardiopulmonary exercise testing and exercise stress echocardiography protocols**

| **Variables** | **CPET(n=90)** | **ESE(n=90)** | ***P* value** |
| --- | --- | --- | --- |
| Workload， W at peak | 123±42 | 119±45 | 0.435 |
| HR beats/minute at peak | 140±19 | 137±18 | ＜0.001 |
| SBP, mm Hg at peak | 174±22 | 179±26 | 0.040 |
| DBP, mm Hg at peak | 85±10 | 85±10 | 0.148 |

HR, heart rate; SBP, systolic blood pressure; DBP, diastolic blood pressure

There were no significant differences in workload and DBP at peak between the two protocols. However, the ESE protocol had a lower HR and a higher SBP at peak, compared with the CPET protocol. It may be related to the different positions where the two exercise protocols were performed, with ESE conducted in a semi-supine position. Regardless of the CPET protocol or the ESE protocol, the same four patients experienced persistent ST segment depression.

**Supplementary Table 3 Reproducibility analysis of echocardiographic parameters**

| **Variable** | **Intra-observer variability** | | **Inter-observer variability** | |
| --- | --- | --- | --- | --- |
|  | **ICC** | **CoV（%）** | **ICC** | **CoV（%）** |
| LVEDV at rest | 0.94（95%CI，0.91-0.96） | 5.5 | 0.93（95%CI，0.90-0.95） | 5.7 |
| LVESV at rest | 0.95（95%CI，0.93-0.97） | 7.8 | 0.92（95%CI，0.89-0.95） | 9.7 |
| SV at rest | 0.93（95%CI，0.90-0.95） | 5.5 | 0.91（95%CI，0.87-0.94） | 6.4 |
| LVGLS at rest | 0.91（95%CI，0.86-0.94） | -3.5 | 0.89（95%CI，0.84-0.93） | -3.8 |
| LVEDV at low load | 0.97（95%CI，0.95-0.98） | 4.3 | 0.96（95%CI，0.94-0.97） | 4.8 |
| SV at low load | 0.98（95%CI，0.97-0.98） | 3.3 | 0.97（95%CI，0.95-0.98） | 3.9 |
| LVEDV at peak | 0.97（95%CI，0.96-0.98） | 4.0 | 0.96（95%CI，0.95-0.98） | 4.6 |
| LVESV at peak | 0.97（95%CI，0.96-0.98） | 8.3 | 0.94（95%CI，0.90-0.96） | 12.5 |
| SV at peak | 0.98（95%CI，0.97-0.98） | 2.9 | 0.95（95%CI，0.93-0.97） | 4.2 |

EDV, end-diastolic volume; ESV, end-systolic volume; SV, stroke volume; LVGLS, left ventricular global longitudinal strain
